# Antiproliferative Effects of the Aptamer d(GGGT)_4_ and Its Analogues with an Abasic-Site Mimic Loop on Different Cancer Cells

**DOI:** 10.3390/ijms23115952

**Published:** 2022-05-25

**Authors:** Antonella Virgilio, Annalisa Pecoraro, Daniela Benigno, Annapina Russo, Giulia Russo, Veronica Esposito, Aldo Galeone

**Affiliations:** Department of Pharmacy, University of Naples Federico II, 80131 Napoli, Italy; antonella.virgilio@unina.it (A.V.); annalisa.pecoraro@unina.it (A.P.); daniela.benigno@unina.it (D.B.); annapina.russo@unina.it (A.R.); giulia.russo@unina.it (G.R.); galeone@unina.it (A.G.)

**Keywords:** guanine-rich oligonucleotides, abasic site mimic, cancer, antiproliferative G-quadruplex

## Abstract

In this paper, we study the T30923 antiproliferative potential and the contribution of its loop residues in six different human cancer cell lines by preparing five T30923 variants using the single residue replacement approach of loop thymidine with an abasic site mimic (S). G-rich oligonucleotides (GRO) show interesting anticancer properties because of their capability to adopt G-quadruplex structures (G4s), such as the G4 HIV-1 integrase inhibitor T30923. Considering the multi-targeted effects of G4-aptamers and the limited number of cancer cell lines tested, particularly for T30923, it should be important to find a suitable tumor line, in addition to considering that the effects also strictly depend on G4s. CD, NMR and non-denaturating polyacrylamide gel electrophoresis data clearly show that all modified ODNs closely resemble the dimeric structure of parallel G4s’ parent aptamer, keeping the resistance in biological environments substantially unchanged, as shown by nuclease stability assay. The antiproliferative effects of T30923 and its variants are tried in vitro by MTT assays, showing interesting cytotoxic activity, depending on time and dose, for all G4s, especially in MDA-MB-231 cells with a reduction in cell viability approximately up to 30%. Among all derivatives, QS12 results are the most promising, showing more pronounced cytotoxic effects both in MDA-MB-231 and Hela cells, with a decrease in cell viability from 70% to 60%. In summary, the single loop residue S substitution approach may be useful for designing antiproliferative G4s, considering that most of them, characterized by single residue loops, may be able to bind different targets in several cancer cell pathways. Generally, this approach could be of benefit by revealing some minimal functional structures, stimulating further studies aimed at the development of novel anticancer drugs.

## 1. Introduction

The fight against cancer is, still today, an ongoing challenge. One of the main difficulties of cancer chemotherapy is eliminating the tumor cells without provoking toxicity in the healthy tissues of the patient. Furthermore, several typical forms of cancer, particularly in their advanced stages, are not sufficiently responsive to traditional chemotherapy agents or may gain endurance to therapy.

It would be necessary to have a novel more specific strategy targeted against cancer cells and less detrimental for the organism than traditional genotoxic plans of chemotherapy. Innovative approaches that work by mechanisms dissimilar from existing therapies, especially those that can target multiple pathways involved in cancer cell survival, may hence be valuable in the treatment of advanced cancer [1]. Quite recently, it was demonstrated that G-rich oligonucleotides (GRO) can show interesting biological properties against different types of tumor cells [2,3,4,5,6]. Repetitive G-rich sequences, commonly expressed in nucleic acids, are able to adopt G-quadruplexes (G4s), typical four-stranded and very stable non-B DNA nucleic acid secondary conformations [7]. The core unit of a G4 is a planar squared arrangement of four guanines (G-tetrad) stabilized by Hoogsteen hydrogen bonds and stacked between them [8]. The secondary structures of DNAs are essential for their function and G4s are widespread in the human genome [9,10], especially in promoter regions and telomere ends [11,12]. Even though the significance of G4 structures in living cells was the subject of controversy in the past, the collection of more recent experimental data corroborates the presence and relevance of these conformations in living cells [13,14]. In addition, the number of reports describing specific proteins able to bind and unfold G4 structures is now considerable, supporting their physiological importance as well [13,14]. Exogenous G-rich oligomers forming stable G4 structures under physiological conditions can recognize these proteins, acting as aptamers. Aptamers are usually created by selecting them from a large random sequence pool through Systematic Evolution of Ligands by EXponential enrichment (SELEX) [15]; however, it is possible to find them in their “natural form” in riboswitches [16]. Aptamers are artificial single-chained DNA or RNA oligonucleotides that are generally 20- to 80-nucleotides-long, which are able to adopt distinctive three-dimensional (3D) structures thanks to several intramolecular interactions [17], such as the very stable nucleic acid secondary conformations G4s in the presence of G-rich sequences and appropriate metal cations. The high binding affinity and specificity of G4-aptamers, among other characteristics, have made them one of the most investigated classes of compounds in diagnostic and therapeutic research areas [18]. One such example is the already known DNA aptamer T30923 (or its partially phosphorothioate version, T30695) [G_3_(TG_3_)_3_T] [19], which was revealed to fold into a dimeric G4, formed by the head-to-head 5′-5′ stacking of two identical propeller-type parallel-stranded G-quadruplex subunits, which is particularly interesting because of its aptitude to target with high affinity two different not related proteins, namely the HIV-1 integrase (HIV-IN) [20,21] and the interleukin-6 receptor (IL-6R) [22].

**Figure 1:**
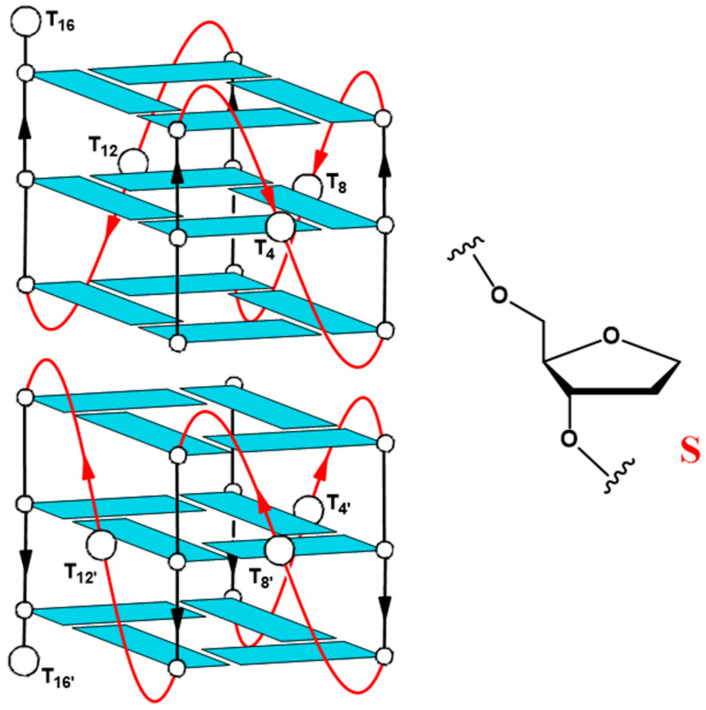
Schematic structure of the parallel-stranded dimeric G-quadruplex Qnat (T30695) and chemical structure of the abasic site mimic (S). Thymidines, replaced by abasic site mimics in the modified sequences, have been labeled.

All guanines in the T30923 sequence are involved in G-tetrad formation and the dimeric parallel-stranded G4 comprises a total of six G-tetrad layers, along with various G-rich oligonucleotides that have HIV-1 integrase inhibition activity, such as T30177 (Zintevir, produced by Aronex Pharmaceuticals in 1996) [23], the first HIV-1 integrase inhibitor tested in clinical trials. Each half of the dimeric quadruplex structure of T30923 is also characterized by three double-chain-reversal loops, protruding outward with respect to the G-tetrad core and particularly relevant for the aptamer/target interaction and for the integrase-inhibiting ability [19,24,25]. In addition to the previously cited antiviral activity, several studies have shown that the T30923 aptamer has also revealed antiproliferative/anticancer properties as well as other G-rich oligonucleotides [2,4,26]. In their paper, Bates et al. described the physical and biological properties of a collection of 12 G4 oligonucleotides, whose structures have formerly been described in depth, and among them T30695 has been proven to possess an intermediate antiproliferative activity on HeLa cervical carcinoma cells that was not very different between NaCl and KCl buffers [2]. Similar G-rich oligonucleotides, namely EAD [T_2_(TG_3_)_4_] [27], ODN S13 [(TG_3_)_3_G_3_] [28], and recently the covalent bi-modular version of the sequence [G_3_(TG_3_)_3_] [29] and T30175 [GTG_2_(TG_3_)_3_T] [30] have been shown to have interesting antiproliferative properties against different cancer cell lines. The G4-aptamer T30923 was also tested for its capability to inhibit Stat3, whose activation has been proven to have an effect on multiple cell fate decisions including proliferation, apoptosis, and differentiation in human cancer cells [24]. The influence of this G4 forming oligodeoxyribonucleotide (G4-STAT3 aptamer) on Stat3 was supported by other studies of Ogloblina et al., which also tested its effect on TOP1 activity [4]. Furthermore, T30923 possesses antiproliferative activity on human breast adenocarcinoma MCF-7 malignant cell line in a dose-dependent manner, but does not affect the viability of immortalized mammary epithelial MCF-10A cells [4]. Considering the multi-targeted effects of G4-aptamers and that only a few cell lines were targeted for studies regarding their antiproliferative activity, particularly for T30923, it should be important to find a suitable cancer cell line, in addition to considering that the effects also strictly depend on the G-quadruplex structure. Hence, in this study, in order to elucidate these two key points, the T30923 antiproliferative potential and the contribution of the residues not belonging to the central core of stacked guanosines were evaluated in different human cancer cell lines. G_3_(TG_3_)_3_T (namely T30923) analogues containing an abasic-site mimic (S) (Figure 1) replacing, one at a time, loop thymidines have been synthesized and studied for their structural and biological properties in comparison to the unmodified original aptamer (Table 1) with the purpose of identifying the G4 features being associated with its biological activity and screening their antiproliferative profile against six different tumor cell lines.

## 2. Results

### 2.1. Structural Insights of the Investigated Sequences

**Figure 2:**
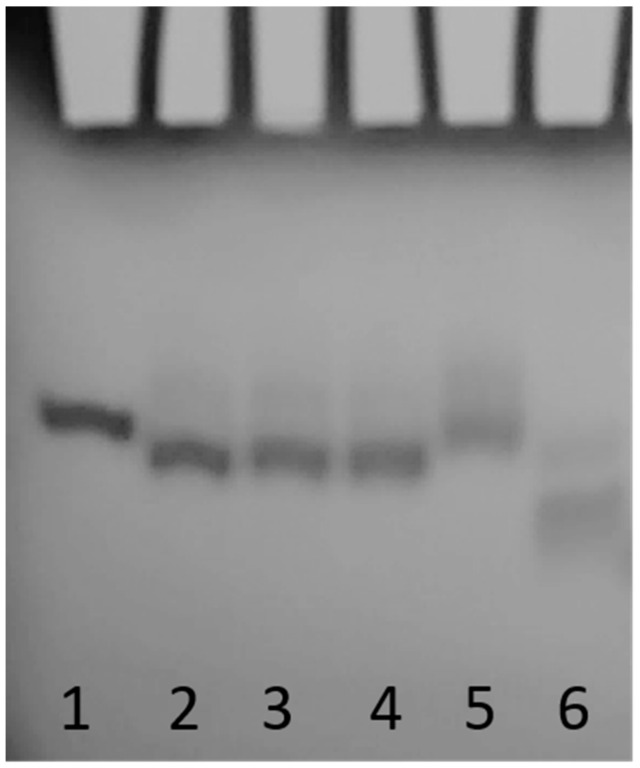
PAGE analysis of the Qnat analogues investigated. Lane 1: Qnat; lane 2: QS4; lane 3: QS8; lane 4: QS12; lane 5: QS16; lane 6: TT-Qnat. See Materials and Methods for experimental details.

As we previously reported [24], the T30923 (Qnat) analogues in Table 1 have been examined by CD and 1H NMR techniques, which are excellent tools to rapidly verify the ability of modified G-rich sequences to adopt G4 structures and to have preliminary information concerning their folding topologies. The modified sequences and their natural counterpart showed CD spectra almost superimposable on each other, exhibiting a minor negative band at 242 nm and a major positive band at 263 nm, typical features of a parallel folding pattern where all guanosines adopt an anti-glycosidic conformation. Furthermore, the registered melting temperatures above 75 °C, as in the case of the original structure, suggested no appreciable influence of the abasic site mimic on the G4 thermal stability. The remarkable similarity of the 1H NMR profiles of the modified ODNs and their unmodified version confirmed that Qnat analogues adopt G4 structures closely comparable with that of the parent one, showing almost superimposable imino proton regions diagnostic of the presence of G-quadruplex structures (10.5–12.0 ppm) [24]. Although these techniques have already allowed us to determine the close topological similarities between the parent ODN and its modified derivatives or testing the possible effects of the abasic site mimic on G4 conformation or stability, no evidence has been provided yet concerning the ability of these modified derivatives to form dimeric G4, as the unmodified Qnat. In order to estimate the molecularity of the G4 complexes, we performed a non-denaturating polyacrylamide gel electrophoresis (PAGE) of all derivatives compared with the natural sequence Qnat, which was proven to form a 5′-5′ dimer of two stacked parallel G4s (Figure 1), and TT-Qnat, corresponding to the natural sequence elongated with two extra thymidines at the 5′-end preventing the formation of the 5′-5′ head-to-head dimer [31] (Figure 2). The migration of the non-denaturated samples of all ODNs containing an abasic site mimic appears to be undoubtedly slower than that of TT-Qnat, which folds mainly into a monomeric G4, and very similar to that of Qnat, thus indicating the presence of dimeric structures also in these cases. In particular, modified ODNs containing an abasic site mimic instead of the loop timidines show slightly faster-migrating bands than Qnat, while the electrophoretic motility of QS16, in which 3′-dT has been substituted by S, is firmly comparable to that of Qnat. These minor differences in migration may be due to slightly more compact structure conformations assumed by the modified sequences which have the S mimic in one of the propeller loops protruding outwards from the G4 central core. In summary, PAGE data confirmed that the modified aptamers can adopt dimeric G4 structures with a strong resemblance to that assumed by the unmodified one.

### 2.2. Nuclease Stability Assay

**Figure 3:**
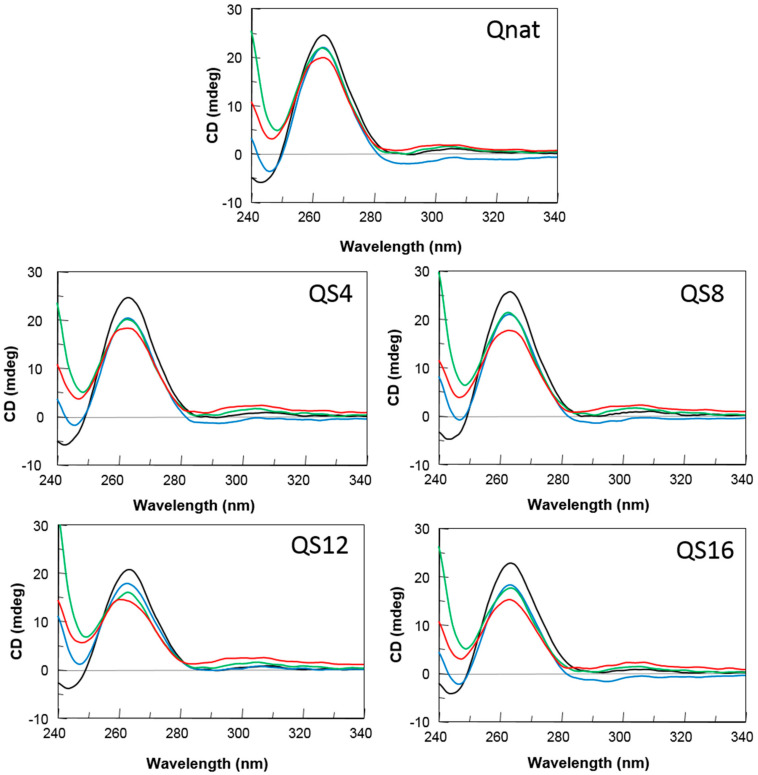
CD spectra of Qnat and its analogues in 10% fetal bovine serum (FBS) diluted with Dulbecco’s Modified Eagle’s Medium (DMEM), registered at 0 (black), 24 (blue), 48 (green), and 72 h (red) at 37 °C. See the main text and the Materials and Methods section for details.

The aptamer susceptibility on nucleases digestion is the major factor limiting the biological effects of oligonucleotides. The introduction of modified residues into an aptamer can influence its biostability. Therefore, to evaluate the resistance in biological environments, all the modified ODNs were incubated in fetal bovine serum (FBS) at 37 °C and analyzed at different time by circular dichroism [32] (Figure 3). In order to verify the persistence over time of the CD signal attributable to undegraded G4 folded species, CD spectra of all ODNs have been registered in 240–340 nm region at 0, 24, 48 and 72 h at 37 °C in 10% FBS. After subtraction of the background scan (10% FBS in DMEM), CD spectra of each sample at different times of incubation preserved the typical CD profile of parallel G4 in which all guanosines adopt anti-glycosidic conformations. Although a time-dependent decrease in band intensities indicated degradation to some extent, a remarkable stability to serum nucleases of Qnat and its abasic mimic derivatives is clearly observable. Particularly, comparing CD spectra at 72 h with those at t_0_, Qnat showed the highest level of nuclease resistance (more than 80% persistent undegraded G4). Nevertheless, its modified derivatives, under the same conditions, also exhibited a noteworthy nuclease resistance since 65–75% of G4 structured species are still present.

### 2.3. Cytotoxic Activity of Qnat and Its Analogues on Different Cancer Cell Lines

**Figure 4:**
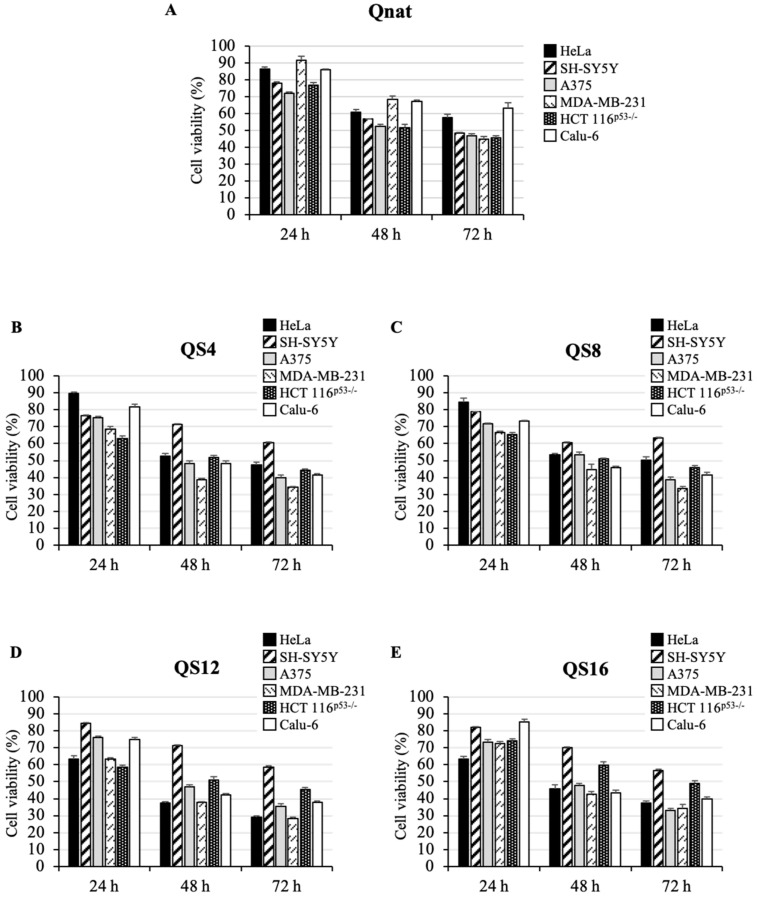
Cytotoxic activity of Qnat (**A**) and its analogues (**B**–**E**) on six different cancer cell lines. Cells were treated with 50 μM of ODNs, from 24 to 72 h. Cell viability was assayed using the MTT assay. Data represent the average of three independent experiments; error bars represent the standard deviation.

To study the cytotoxic activity of the investigated ODNs, an MTT assay was performed in different human tumor cell lines, such as HeLa (cervical carcinoma), SH-SY5Y (neuroblastoma), A375 (melanoma), MDA-MB-231 (breast cancer), HCT 116^p53−/−^ (colon cancer) and Calu-6 (lung cancer). To this aim, cells were treated with two concentrations of ODNs, 10 μM (Figure S1) and 50 μM (Figure 4) from 24 to 72 h. Our results show that Qnat and its analogues consistently inhibited cell proliferation in all tested tumor cell lines in a time- and dose-dependent manner (Figure 4 and Figure S1), since, in most cases, evident effects on the cell viability were recorded after 72 h at 50 μM. However, at this ODN concentration, Qnat in A375 and HCT 116^p53-/-^ cells reduced cell viability by 50% already after 48 h and retained this effect even at 72 h, whereas in SH-SY5Y and MDA-MB-231 cells, the same cytotoxic effect was obtained only after 72 h (Figure 4A).

Notably, Qnat analogues showed a higher cytotoxic activity than the unmodified original aptamer, in almost all cases, apart from the SH-SY5Y cell line. The most pronounced cytotoxic effects were found after 72 h at 50 μM in A375, MDA-MB-231 and Calu-6 cell lines for QS4 and QS8, instead in A375, MDA-MB-231 and Hela for QS12 and QS16. At the same time point, a reduction in cell viability from 50% in Qnat treated cells to 30–40% in the analogues treated A375 and MDA-MB-231 cells was observed; while, in Calu-6 cells, a decrease in cell viability from 65% in Qnat-treated cells to 40% in Qnat derivatives treated cells was found.

Interestingly, Qnat analogues exerted a different cytotoxic effect in HeLa cells. In particular, QS4 and QS8 (Figure 4B,C) showed a cytotoxic activity slightly higher than Qnat, whereas QS12 and QS16 exhibited more pronounced cytotoxic effects, especially QS12 which proved to reduce the cell viability of ∼70% after 72 h of treatment at 50 μM (Figure 4D,E).

Finally, all Qnat analogues showed a cytotoxic activity on HCT 116^p53^^−/^^−^ cell line comparable to that of Qnat, suggesting that the single T residue replacement with an abasic site mimic in T30923 sequence does not have a significative effect in this case (Figure 4), on the contrary this chemical modification significantly affects the cytotoxic activity on SH-SY5Y cell line since the unmodified aptamer is the most active on these cancer cells.

It is noteworthy that the cytotoxic activity of the investigated ODNs on normal human lung fibroblast cell line (MRC-5 fibroblast cell line) was drastically reduced compared to that observed in cancer cells. In particular, all ODNs reduced only the 30% of cell viability of MRC-5 cells after 72 h of treatment at the highest tested dose with a comparable pattern (Figure S2).

## 3. Discussion

The relevance of G4 structure formation and stabilization as a therapeutic approach to treat cancer cells has been well known for many decades, considering that the clinical use of anticancer drugs is limited by their general toxicity to proliferating cells and low selectivity [2]. The G4 structures adopted by GROs are mostly very stable because they are characterized by a central core of stacked G-tetrads (two to four usually), whereas the loops, being projected outwardly, are the aptamer portions mostly implicated in the interaction with the protein target [20,21,22,23,24]. Therefore, a smart way of rationally designing an aptamer is to disclose its minimum functionality requirements and work on them. To achieve this, by using the single residue replacement approach, we have produced five T30923 variants with the purpose of exploiting the role and function of loop in the viability inhibition of different cancer cell lines. CD, NMR and non-denaturating PAGE data clearly showed that all ODNs investigated maintain the original ability of T30923 to fold in 5′-5′ end-to-end stacked dimers of parallel G4 structures, closely resembling that of the unmodified aptamer. Their resistance in biological environments was tested by a nuclease stability assay in fetal bovine serum (FBS) at 37 °C at different times. The S derivatives persist as undegraded G4 structures at 72 h in a range from 65 to 75%, while the Qnat exhibits more than 80% of nuclease resistance, showing that a single T loop substitution with a S residue does not substantially affect the aptamers’ susceptibility on nucleases digestion, which is one of the main aspects limiting the biological efficacy of oligonucleotides. To determine whether Qnat and its derivatives affected cell growth in vitro, we have analyzed, by a MTT assay, their cytotoxic effects against six different cancer cell lines, namely cervical carcinoma (HeLa), neuroblastoma (SH-SY5Y), melanoma (A375), breast cancer (MDA-MB-231), colon cancer (HCT 116^p53−/−^) and lung cancer (Calu-6) cells. For this purpose, cells were incubated with ODNs for 24, 48, and 72 h at two different concentrations, 10 μM (Figure S1) and 50 μM (Figure 4). Qnat and all analogues showed an interesting cytotoxic activity, depending on time and dose. It is noteworthy that the unmodified aptamer is the most efficient in inhibiting SH-SY5Y cell growth since it is the only one able to reduce the viability of these cells by 50%, suggesting that in this cancer cell line the presence of all T residues of Qnat is an important feature for preserving its biological properties. In all other evaluated human tumor cell lines, the abasic site mimic containing derivatives showed an antiproliferative effect that was more pronounced than the parent aptamer, probably accounting for a favorable contribution to biological activity of the abasic region in the structure. Specifically, the MDA-MB-231 cells are the most affected by T30923 analogues, with a reduction in cell viability approximately up to 30% at 72 h. These results are particularly interesting considering that this tumor cell line is a highly aggressive, invasive, and poorly differentiated triple-negative breast cancer (TNBC) category [33,34]. Among all derivatives, QS12 seems to be the most promising one since it shows a more pronounced cytotoxic effect both in MDA-MB-231 and HeLa cancer lines, especially with a decrease in cell viability from 70% to 60% after 72 h of treatment at 50 μM.

## 4. Materials and Methods

### 4.1. Oligonucleotide Synthesis and Purification

Oligonucleotides reported in Table 1 were synthesized by an ABI 394 DNA synthesizer using solid phase β-cyanoethyl phosphoramidite chemistry at 10 µmol scale. The synthesis was performed using normal 3′-phosphoramidites and a 5′-dimethoxytrityl-3′-phosphoramidite-1’,2’-dideoxyribose (dSpacer, S, Link Technologies, Glasgow, UK) for the introduction of an abasic site mimic moiety in each sequence. For ODN QS16, a universal support was used. The oligomers were detached from the support and deprotected by treatment with concentrated aqueous ammonia at 80 °C overnight. The combined filtrates and washings were concentrated under reduced pressure, redissolved in H_2_O, analyzed and purified by high-performance liquid chromatography on a Nucleogel SAX column (Macherey–Nagel, Duren, Germany, 1000-8/46), using buffer A: 20 mM KH_2_PO_4_/K_2_HPO_4_ aqueous solution (pH 7.0) containing 20% (*v*/*v*) CH_3_CN and buffer B: 1 M KCl, 20 mM KH_2_PO_4_/K_2_HPO_4_ aqueous solution (pH 7.0) containing 20% (*v*/*v*) CH_3_CN; a linear gradient from 0 to 100% B for 45 min and flow rate 1 mL/min were used. The fractions of the oligomers were collected and successively desalted by Sep-pak cartridges (C-18). The isolated oligomers proved to be >98% pure by NMR (500 MHz, D_2_O, 80 °C) (Figure S3) and HPLC (Macherey–Nagel, 1000-8/46, buffer A: 20 mM NaH_2_PO_4_/Na_2_HPO_4_ aqueous solution (pH 7.0) containing 20% (*v*/*v*) CH_3_CN; buffer B: 1 M NaCl, 20 mM NaH_2_PO_4_/Na_2_HPO_4_ aqueous solution (pH 7.0) containing 20% (*v*/*v*) CH_3_CN; linear gradient from 0 to 100% B for 45 min and flow rate 1 mL/min) (Figure S4).

### 4.2. Gel Electrophoresis

All oligonucleotides were analyzed by non-denaturing PAGE. Samples of modified oligonucleotides and their natural counterpart were prepared at a ODN concentration of 1mM by using a potassium phosphate buffer (10 mM KH_2_PO_4_/K_2_HPO_4_, 70 mM KCl, pH 7.0) and submitted to the annealing procedure (heating at 90 °C and slowly cooling at room temperature). Each oligonucleotide was loaded on a 20% polyacrylamide gel containing Tris–Borate-EDTA (TBE) 2.5x and KCl 20 mM. The run buffer was TBE 1× containing 50 mM KCl. For all samples, a solution of glycerol/TBE 10× was added just before loading. Electrophoresis was performed at 8 V/cm at a temperature close to 10 °C. Bands were visualized by UV shadowing.

### 4.3. Nuclease Stability Assay

Nuclease stability assay of all ODNs was conducted in 10% Fetal Bovine Serum (FBS) diluted with Dulbecco’s Modified Eagle’s Medium (DMEM) at 37 °C and studied by CD analysis. Approximately 7 nmol of stock solution of each ODN (~1 O.D.U.) was evaporated to dryness under reduced pressure and then incubated with 250 μL 10% FBS at 37 °C. The degradation patterns were analyzed by monitoring the CD signal decrease in each sample at 37 °C, as a function of time. CD spectra at 0, 24, 48 and 72 h for all ODNs were recorded at 37 °C using a Jasco 715 spectrophotometer equipped with a Peltier temperature control system (Jasco, Tokyo, Japan). Data were collected from 240 to 320 nm with a 1 s response time and a 1 nm bandwidth using a 0.1 cm quartz cuvette. Each spectrum shown is corrected for the spectrum of the reaction medium (10% FBS in DMEM).

### 4.4. Cell Cultures and Treatments with the ODNs

HeLa, SH-SY5Y, A375, MDA-MB-231, HCT 116p53^−/−^, Calu-6 and MRC-5 cell lines were cultured as previously reported [35]. Briefly, cells were cultured in Dulbecco’s modified Eagle’s medium (DMEM), supplemented with 10% fetal bovine serum (FBS), 2 mM L-glutamine and 50 U/mL penicillin-streptomycin, under a humidified atmosphere of 5% CO_2_ at 37 °C. Treatments of cells were performed replacing the culture medium with those containing ODNs at final concentration of 10 μM and 50 μM.

### 4.5. MTT Assay

Cells were seeded onto 96-well plates at a density of 0.5 × 104 cells/well and treated with different ODNs at final concentration of 10 μM and 50 μM from 24 h to 72 h. Then, cell viability was determined using the MTT assay as previously reported [36]. A pool of three different sets of experiments each repeated in triplicate were performed. Error bars represent the standard deviation. Statistical comparisons were made as previously shown [37].

## 5. Conclusions

In summary, we have investigated a small library of T30923 variants with the aim to exploit the role and function of loop in the viability inhibition of different cancer cell lines. T30923 and all derivatives showed interesting cytotoxic activity, depending on time and dose, especially in MDA-MB-231 cells with a reduction in cell viability approximately up to 30%. The most promising one was QS12 showing more pronounced cytotoxic effects both in MDA-MB-231 and Hela cells, with a decrease in cell viability from 70% to 60%.

Our results suggest that the single loop residue abasic substitution may represent a useful route for the design of antiproliferative G4-oligonucleotides, in particular taking into account that several G4 aptamers endowed with antiproliferative activity, binding different targets and therefore being involved in different cancer cell pathways, are characterized by loops containing a single residue, such as AS1411 and anti-STAT aptamer [26,38]. Generally, this approach could be beneficial, as it reveals some minimal functional structures and manipulates them, thus stimulating further studies aimed at the development of novel anticancer drugs.

## Figures and Tables

**Table 1:** Names and sequences of Qnat (T30923) and its analogues. **S** indicates an abasic site mimic.

Oligonucleotide	Sequence
Qnat	5′-GGGTGGGTGGGTGGGT-3′
QS4	5′-GGG**S**GGGTGGGTGGGT-3′
QS8	5′-GGGTGGG**S**GGGTGGGT-3′
QS12	5′-GGGTGGGTGGG**S**GGGT-3′
QS16	5′-GGGTGGGTGGGTGGG**S**-3′

## Data Availability

Data is contained within the article or Supplementary Materials.

## Supplementary Materials

The following supporting information can be downloaded at: https://www.mdpi.com/article/10.3390/ijms23115952/s1.
