# Supplementary material for: Antiproliferative Effects of the Aptamer d(GGGT)4 and Its Analogues with an Abasic-Site Mimic Loop on Different Cancer Cells
Source: Int J Mol Sci. 2022 May 25;23(11):5952. doi: 10.3390/ijms23115952 (PMC9181107; doi:10.3390/ijms23115952)
Supplement: Supplementary file 1 [file ijms-23-05952-s001.zip › ijms-1716536-supplementary.pdf]

Supplementary Material

Antiproliferative effects of the aptamer d(GGGT)4 and its analogues with an abasic-site mimic loop on different cancer cells.

Antonella Virgilio, Annalisa Pecoraro, Daniela Benigno, Annapina Russo, Giulia Russo, Veronica Esposito* and Aldo Galeone

Department of Pharmacy, University of Naples Federico II, Napoli, Italy.
*Correspondence: Corresponding author: VE: verespos@unina.it.

Table of contents

MTT assay of Qnat and its analogues at 10 μM of ODNs
Cytotoxic activity of Qnat and its analogues on healthy cell line
1H-NMR spectra of purified oligomers in D_2O at 80°C
HPLC traces of Qnat and its analogues

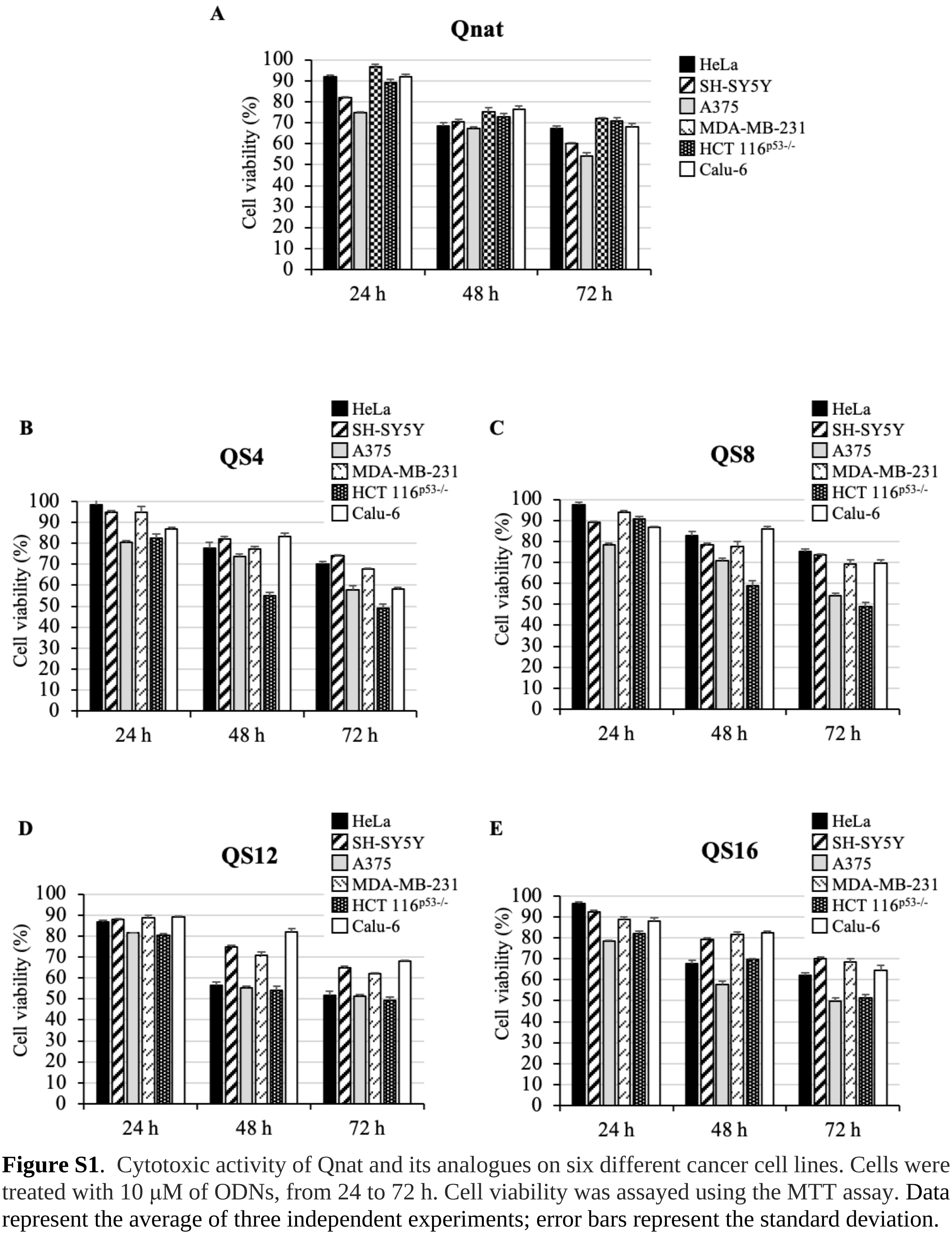

Figure S1. Cytotoxic activity of Qnat and its analogues on six different cancer cell lines. Cells were treated with 10 μM of ODNs, from 24 to 72 h. Cell viability was assayed using the MTT assay. Data represent the average of three independent experiments; error bars represent the standard deviation.

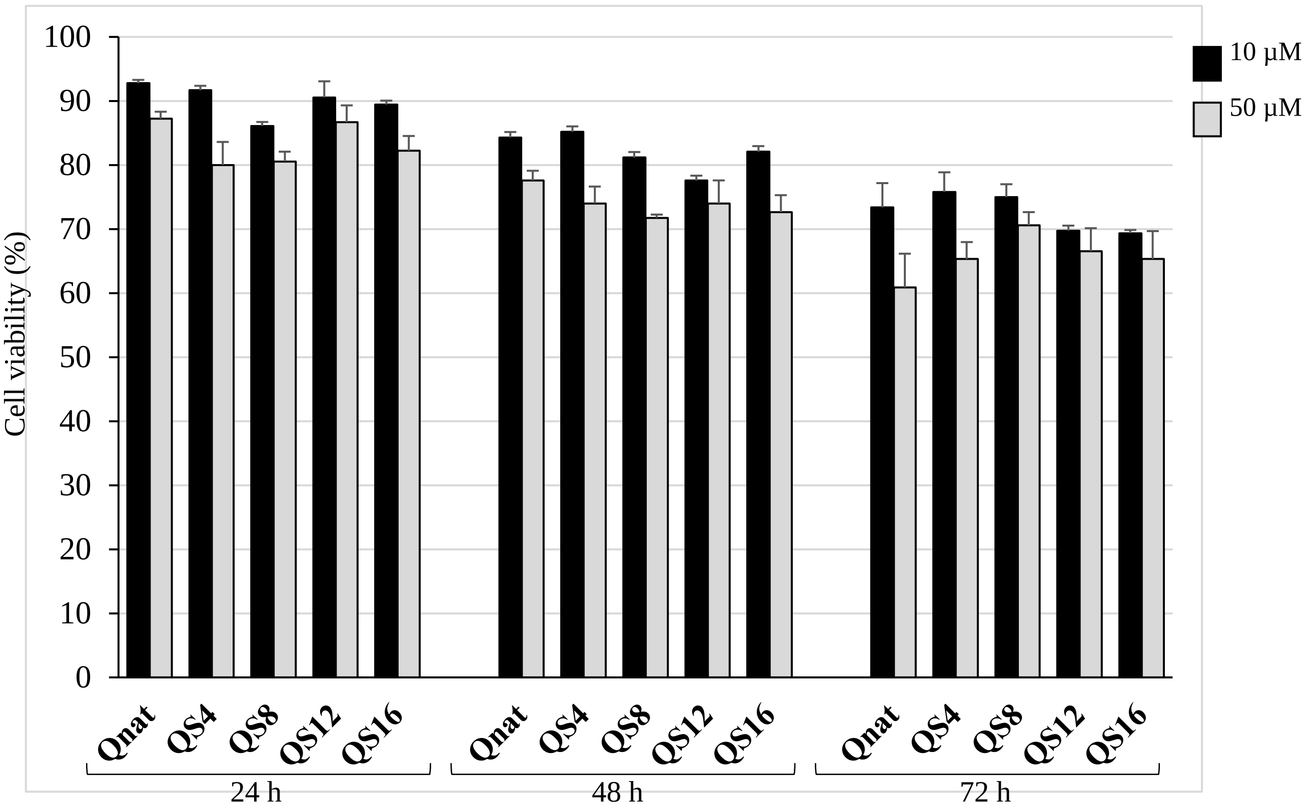

Figure S2. Cytotoxic activity of Qnat and its analogues on MRC-5 cell line. Cells were treated with 10 µM and 50 µM of ODNs, from 24 to 72 h. Cell viability was assayed using the MTT assay. Data represent the average of three independent experiments; error bars represent the standard deviation.

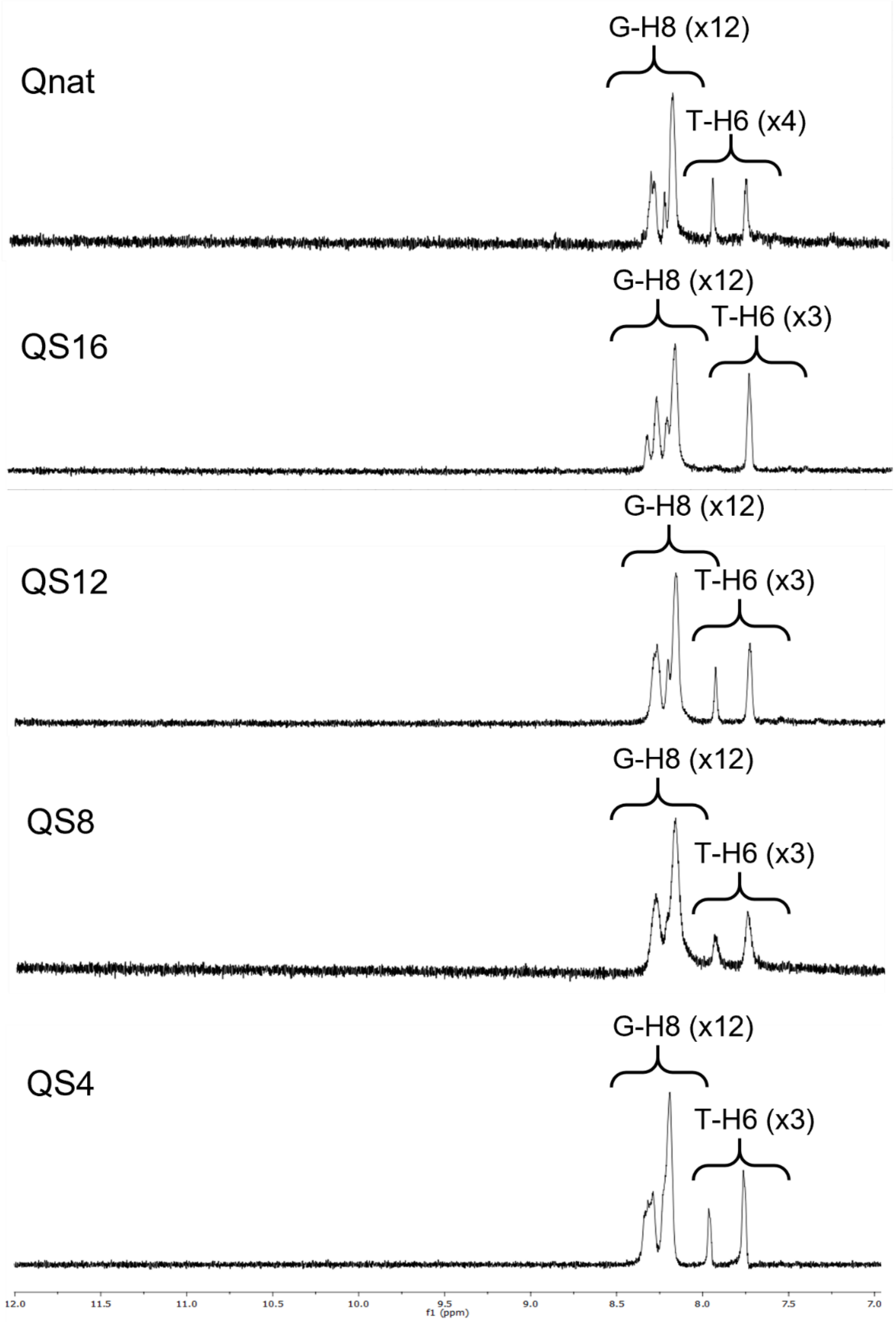

Figure S3. Aromatic region of the high resolution NMR spectra (700 MHz, D_2O, 80°C, no salt) of purified oligomers.

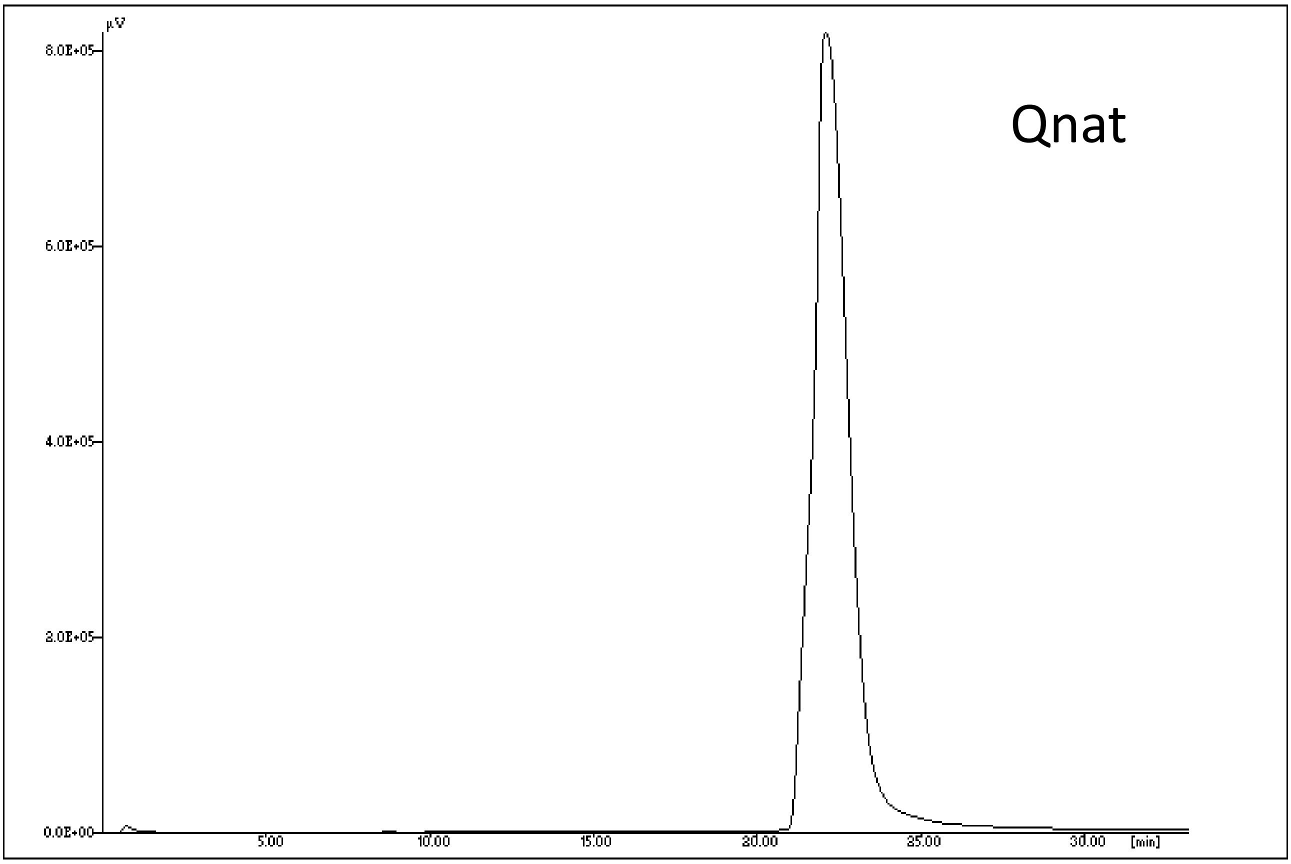

Figure S4. HPLC chromatograms of Qnat and its analogues. See experimental section for details

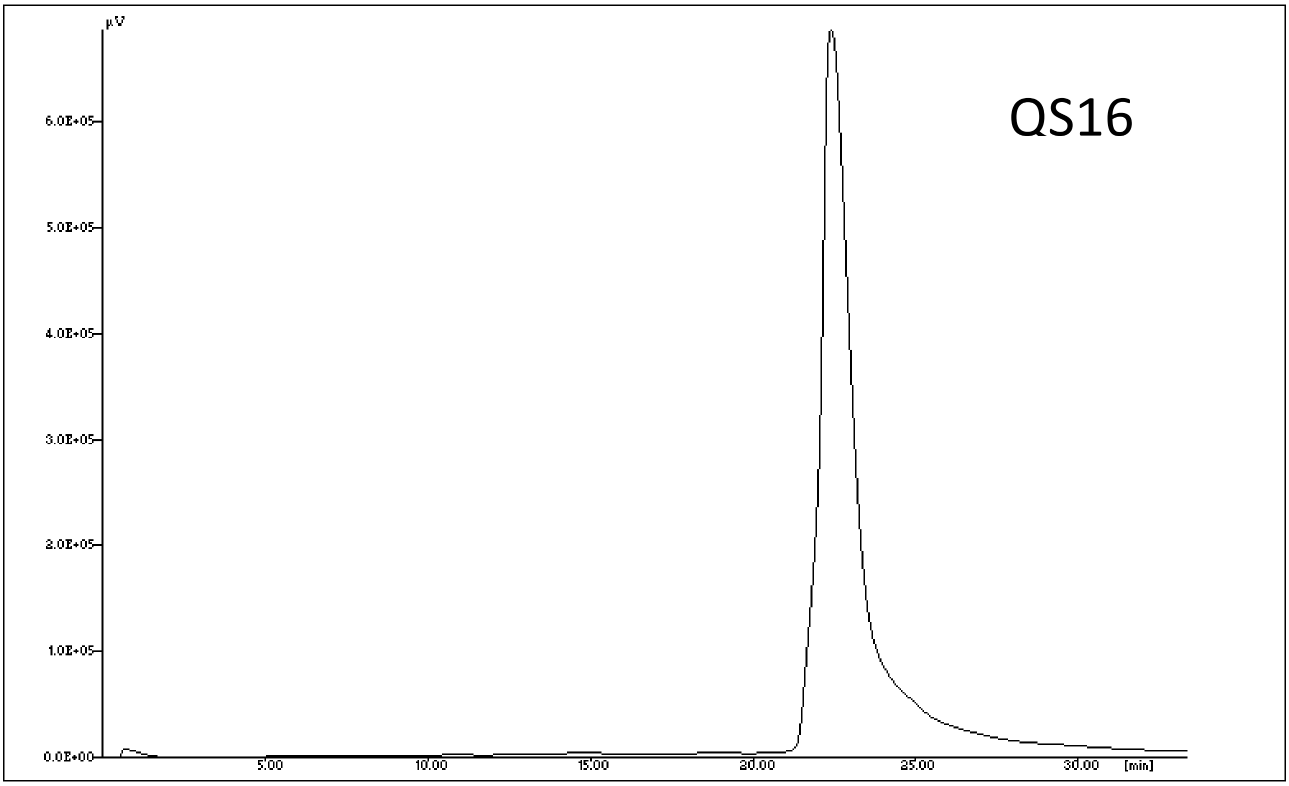

Figure S4. *Cont.*

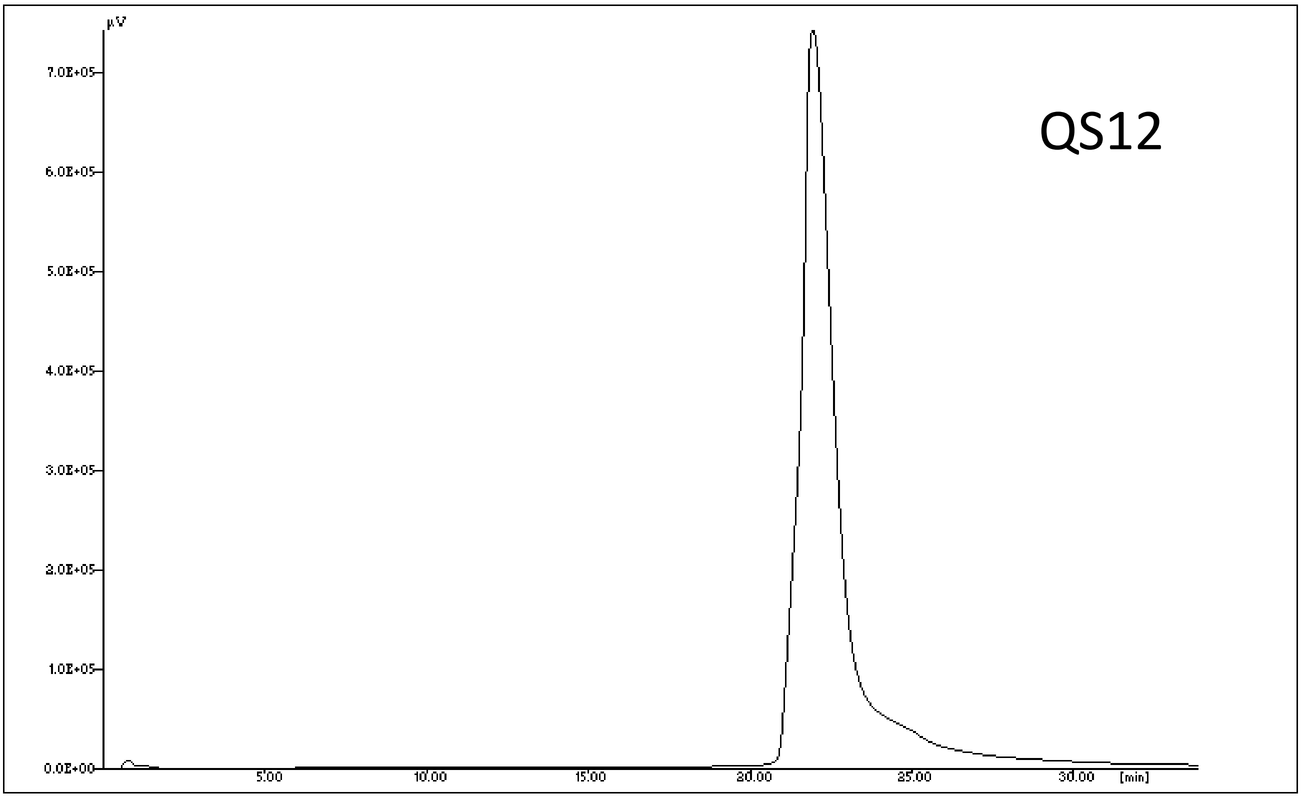

Figure S4. *Cont.*

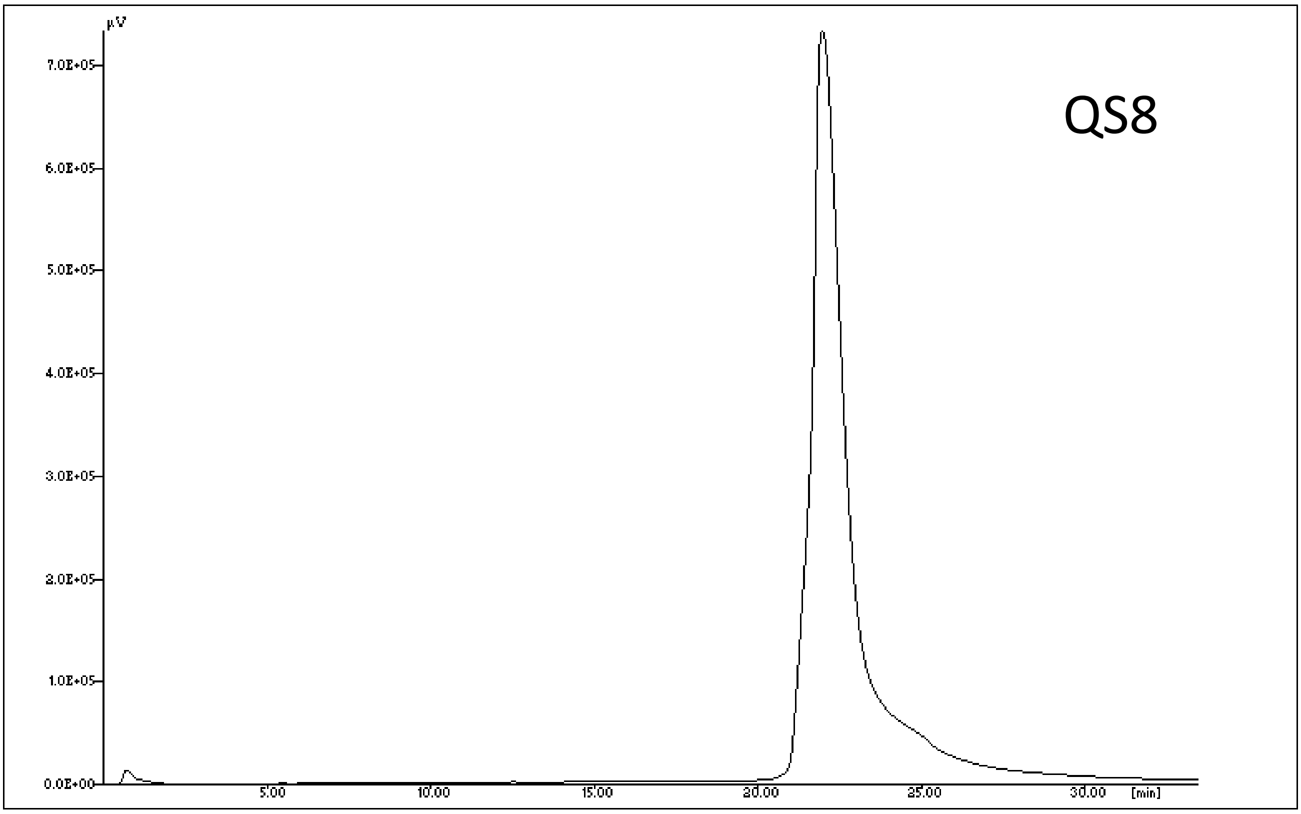

Figure S4. *Cont*

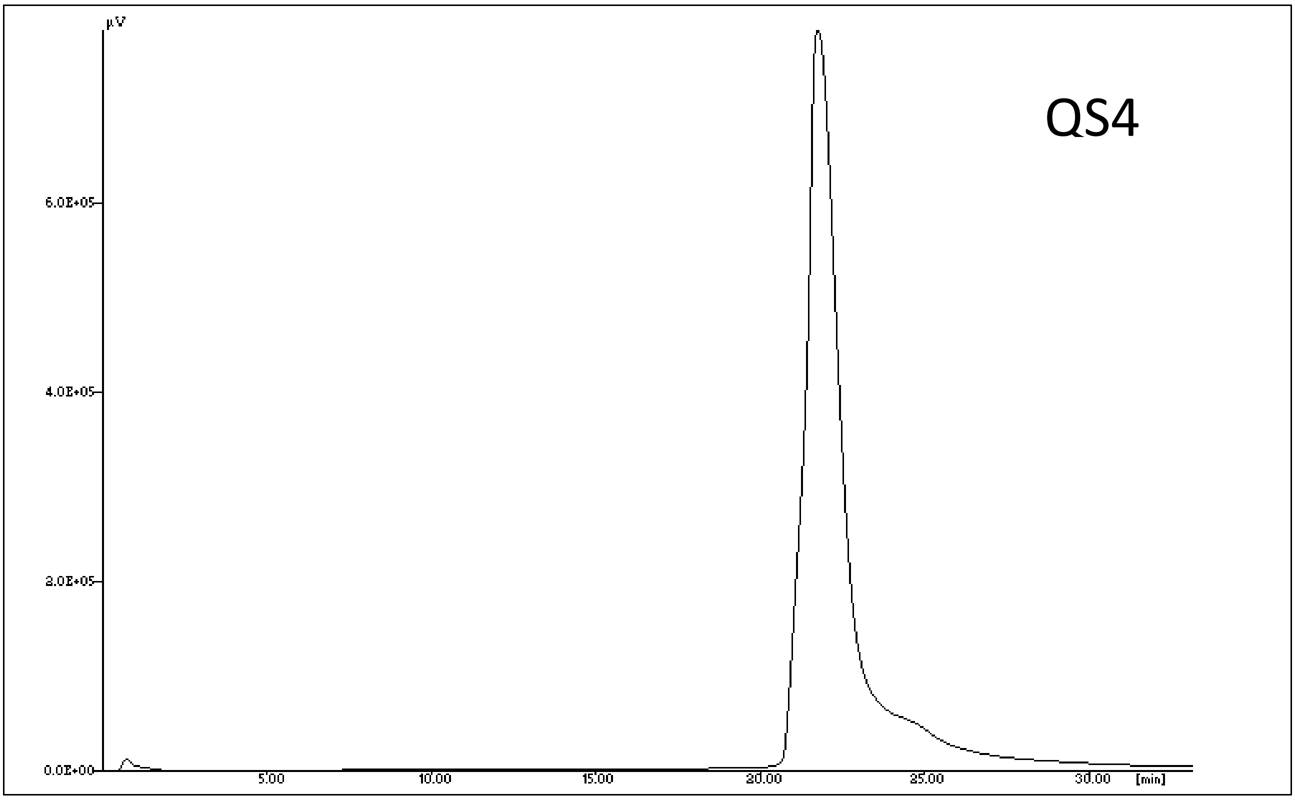

Figure S4. *Cont*
